# A Treatise on the Science and Practice of Midwifery

**Published:** 1899-12

**Authors:** 


**A Treatise on the Science and Practice of Midwifery.** By W. S. Playfair, M.D. Ninth Edition. Two volumes. Pp. xviii., 446; xv., 454. London: Smith, Elder, & Co. 1898.

Evidence of the popularity of this standard work is proved by the fact that yet another edition is added to those which have already appeared. Its popularity is easily understood. It is written in a very taking style, is well illustrated, and is sufficiently dogmatic to be impressive to the student. The last edition brings it into line with the latest research work done on the subject, and contains many new and valuable facts.

The description of the structure and function of the placenta is based upon the recent work done by Dr. Eden, and gives several interesting and important observations carried out by him. The subject is handled in a masterly manner. The management of labour and its complications is dealt with as in previous editions, and needs little comment: the author's well-known sound teaching and correct deductions leave little scope for the critic.

Two extremely interesting additions are introduced: one is the account of the death of the Princess Charlotte—a most impressive picture of the dangers of labour unduly prolonged by reluctance of the attendants to interfere. The other is an excellent criticism of a paper by Dr. Japp Sinclair, who ascribed the present surgical aspect of gynæcology largely to defective operative midwifery practice. Dr. Playfair brings some excellent arguments to show that Dr. Sinclair's views are at least exaggerated.

We notice that Dr. Playfair omits to mention the use of veratrum viride in eclampsia, and rather discourages forced delivery in these cases. His views on the latter subject are powerfully opposed by several first-class authorities, and the use of the drug named has been shown to be valuable.

There is one point to which we would take exception: Dr. Playfair advises that uterine irrigation should be carried out by means of a Higginson's syringe. We regard this as a most unfortunate piece of advice, as that instrument is likely to inject air, is difficult to clean, and necessitates the use of two hands; it is an inefficient, dirty, and dangerous instrument, fit only for the original purpose for which it was devised. Gravitation is the only sound method of uterine irrigation. Hayes's tube, which Dr. Playfair also recommends for intra-uterine injections, has the great defect of having no provision for the passage of the outflowing current from the uterus.

